# Supplementary material for: A Metric-Based, Meta-Analytic Appraisal of Environmental Enrichment Efficacy in Captive Primates
Source: Animals (Basel). 2025 Mar 11;15(6):799. doi: 10.3390/ani15060799 (PMC11939658; doi:10.3390/ani15060799)
Supplement: Supplementary file 1 [file animals-15-00799-s001.zip › Duncan&Pillay_TableS1.pdf]

<u>Supplementary: Table S1</u>. Distribution of research protocols across peer-reviewed journals used in the meta-analysis of captive primate environmental enrichment

Journal	Protocols
American Journal of Primatology	33
Animals	3
Animal Behaviour	2
Animal Behavior and Cognition	1
Animal Welfare	26
Anthrozoös	1
Applied Animal Behaviour Science	57
Brain Research	1
Infant Behavior and Development	1
International Journal of Primatology	9
International Zoo Yearbook	4
Journal of the American Association for Laboratory Animal Science	8
Journal of Applied Animal Welfare Science	30
Journal of Medical Primatology	7
Malayan Nature Journal	1
Neotropical Primates	1
Physica A	1
Primates	6
Reproductive Toxicology	1
Zoo Biology	54
Zoologia	1
	248
