# Supplementary material for: A Metric-Based, Meta-Analytic Appraisal of Environmental Enrichment Efficacy in Captive Primates
Source: Animals (Basel). 2025 Mar 11;15(6):799. doi: 10.3390/ani15060799 (PMC11939658; doi:10.3390/ani15060799)
Supplement: Supplementary file 1 [file animals-15-00799-s001.zip › Duncan&Pillay_TableS2.pdf]

Supplementary: Table S2. *Summary table of enrichment efficacy scores (x̄ ± SE) for each enrichment type per species as identified in the literature. Values in brackets represent the number of published protocols per condition.*

Family	Species	Feeding	Object	Sensory			Social	Cognitive	Training	Interaction-based	Enclosure modification	Combination	Other
				Olfactory	Auditory	Visual							
Galagidae	*Galago senegalensis*	0.50 (1)	-	0.00 (1)	-0.50 (1)	-	-	-	-	-	-	-	-
Lorisidae	*Nycticebus bengalensis*	0.39 (1)	-	-	-	-	-	-	-	-	-	-	-
	Nycticebus coucang	0.39 (1)	-	-	-	-	-	-	-	-	-	-	-
	Nycticebus pygmaeus	-	-	-	-	-	0.60 ± 0.40 (2)	-	-	-	-	-	-
	Perodicticus potto	0.50 (1)	-	-	-	-	-	-	-	-	1.00 (1)	-	-
Lemuridae	*Eulemur fulvus*	0.24 ± 0.10 (2)	-	-	-	-	-	-	-	-	-	-	-
	Hapalemur griseus	0.67 (1)	-	-	-	-	-	-	-	-	-	-	-
	Lemur catta	0.20 (1)	-	0.25 (1)	-	-	-	-	0.06 (1)	-	-	-	-
	Varecia variegata	0.33 (1)	-	-	-	-	-	-	-	-	-	-	-
Callitrichidae	*Callimico goeldii*	-	-	-	-	-	-	-	-	-	-	0.00 (1)	0.00 (1)
	Callithrix geoffroyi	-	0.00 (1)	-	-	-	-	-	-	-	-	-	-
	Callithrix jacchus	0.08 ± 0.08 (3)	-	-	-	-	-	-	-	0.27 (1)	0.51 ± 0.16 (6)	-	-
	Callithrix penicillata	-	-	-	-	-	-	-	-	-	-	1.00 (1)	-
	Cebuella pygmaea	-	0.00 (1)	-	-	-	-	-	-	-	-	-	0.00 (1)
	Leontopithecus chrysomelas	-	0.00 (1)	-	-	-	-	-	-	-	-	-	-
	Leontopithecus rosalia	0.15 (1)	-	-	-	-	-	-	-	-	-	-	-
	Saguinus labiatus	-	0.00 (1)	-	-	-	-	-	-	-	-	-	-
	Saguinus oedipus	-	-	-	-	-	-0.08 ± 0.22 (3)	-	-	-	0.04 ± 0.04 (4)	-	0.38 ± 0.13 (2)
Cebidae	*Cebus capucinus*	0.25 (1)									0.15 (1)	-	-
	Saimiri boliviensis	0.67 (1)	-	-	-	-	-	-	-	-	-	-	-
	Saimiri sciureus	0.00 (1)	-	-	-	-	0.03 (1)	-	-	-	0.33 (1)	0.00 ± 0.00 (2)	0.00 (1)
	Sapajus apella	0.52 ± 0.24 (3)	0.21 ± 0.07 (3)	-	-	-	-	-	0.67 (1)	-	0.35 ± 0.15 (2)	0.25 ± 0.03 (3)	0.00 (1)
	Sapajus nigritus	1.00 (1)	-	-	-	-	-	-	-	-	-	-	-
	Sapajus xanthosternos	-	-	-	-	-	-	-	-	-	0.05 (1)	-	-0.08 (1)
Cercopithecidae	*Cercocebus galeritus*	-	-	-	-	-	-0.17 ± 0.17 (2)	-	-	-	0.17 ± 0.50 (2)	-	-
	Cercocebus torquatus	0.20 ± 0.20 (2)	-	-	-	-	-	-	-	-	0.06 ± 0.10 (3)	0.08 (1)	-
	Cercopithecus mona	0.14 (1)	-	-	-	-	-	-	-	-	-	-	-
	Cercopithecus wolfi	-	-	-	-	-	-	-	-	-	0.29 (1)	-	-
	Chlorocebus aethiops	-	-	-	0 (1)	-	-	-	-	-	-	-	-
	Colobus guereza	-	-	-	-	-	-	-	-0.22 (1)	-	-	-	-
	Lophocebus albigena	0.40 (1)	-	-	-	-	-	-	-	-	0.11 (1)	-	-
	Macaca arctoides	0.19 ± 0.31 (2)	-0.31 ± 0.06 (2)	-	-	-	0.00 (1)	-	-	-	-	0.83 (1)	0.25 (1)
	Macaca fascicularis	-	-	-	-	-	0.05 ± 0.05 (2)	-	-	-	-0.43 (1)	0.09 (1)	-
	Macaca fuscata	-	-	-	-	0.50 ± 0.25 (3)	-	-0.22 (1)	-	-	0.21 ± 0.21 (2)	-	-
	★*Macaca mulatta*	0.50 ± 0.09 (7)	0.25 ± 0.14 (4)	-	-	0.00 (1)	0.37 ± 0.06 (9)	0.00 (1)	0.25 ± 0.25 (2)	-	0.05 ± 0.03 (4)	0.22 ± 0.09 (11)	0.14 ± 0.07 (3)
	Macaca nemestrina	-	0.15 ± 0.15 (2)	-	-	-	-	-	-	-	-	-	-
	Macaca nigra	-	-	-	-	-	-	-	1.00 (1)	-	0.19 (1)	-	-
	Macaca radiata	0.00 (1)	-	-	-	-	-	-	-	-	-	-	-
	Macaca silenus	-	-	-	-	-	0.60 (1)	-	-	-	0.33 (1)	0.33 (1)	-
	Macaca sylvanus	-0.25 (1)	-0.19 ± 0.06 (2)	-	-	-	-	-	-	-	-	-	-
	Mandrillus leucophaeus	0.28 (1)	-	-	-	-	-	-	-	-	-	-	-0.33 (1)
	Mandrillus sphinx	-	-	-	-	-	-	0.50 (1)	-	-	-	0.17 (1)	-
	Papio anubis	-	-	-	-	-	0.00 (1)	-	-	-	-	-	-
	Papio hamadryas	0.00 (1)	-	-	0.10 (1)	-	-	-	-	-	0.13 (1)	0.54 (1)	-
	Papio papio	-	-	-	-	-	-	0.43 (1)	-	-	-	-	-
	Semnopithecus priam	-	-	-	-	-	-	-	-	-	-	0.54 (1)	-
Hylobatidae	*Hylobates lar*	0.14 (1)	-	-	0.33 (1)	-	-	-	-	-	-	-	-
	Hylobates moloch	0.06 ± 0.06 (2)	0.13 (1)	0.13 (1)	-	-	-	-	-	-	-	-	-
	Hylobates pileatus	-	-	-	-	-	-	-	-	-	-	0.33 (1)	-
	Nomascus leucogenys	-	-	-	-	-	-	-	-	-	1 (1)	-	-
	Symphalangus syndactylus	-	-	-	-	-	-	-	-	-1.00 (1)	1.00 (1)	-	-
Hominidae	*Gorilla gorilla*	0.32 ± 0.13 (3)	0.07 (1)	0.08 (1)	0.08 ± 0.17 (2)	-	0.11 ± 0.11 (2)	0.75 (1)	0.08 (1)	-	0.19 ± 0.08 (4)	-	-0.08 (1)
	Pan paniscus	0.67 (1)	-	-	-	-	0.09 (1)	-	-	-	-	-	-
	Pan troglodytes	0.23 ± 0.09 (6)	-	-	-	0.00 ± 0.00 (2)	0.23 ± 0.15 (3)	0.33 ± 0.19 (5)	0.31 ± 0.34 (4)	0.38 (1)	0.12 ± 0.07 (5)	0.33 (1)	0.32 ± 0.22 (5)
	Pongo pygmaeus	0.43 ± 0.18 (2)	-	-	-	-	0.00 ± 0.00 (2)	-0.40 (1)	-	-	-	0.50 (1)	0.00 (1)

★ *One protocol did not explicitly describe the nature of the enrichment used and was thus not included here.*
