# Supplementary material for: A Metric-Based, Meta-Analytic Appraisal of Environmental Enrichment Efficacy in Captive Primates
Source: Animals (Basel). 2025 Mar 11;15(6):799. doi: 10.3390/ani15060799 (PMC11939658; doi:10.3390/ani15060799)
Supplement: Supplementary file 1 [file animals-15-00799-s001.zip › Duncan&Pillay_TableS3.pdf]

Supplementary: Table S3. Taxonomic summary of the literature relating to captive primate environmental enrichment. Sample sizes are reported for the enrichment protocols. Incomplete sample sizes (i.e. species with undisclosed sample sizes) are indicated with a star (★). Efficacy scores represent an index ranging from -1 to 1, corresponding to an absolute deterioration and improvement in animal welfare respectively for all measures reported. An efficacy score of 0 implies no change in animal welfare status for all measures considered. For species where only a single protocol currently exists, the efficacy score value presented is the efficacy score for that protocol alone and not a mean value for the species.

		Number of protocols		Sample sizes		Mean efficacy score ($\bar{x}$ ± SE)	
**Family**	**Species**	**Per species**	**Per family**	**$\bar{x} \pm s$**	**Total**	**Per species**	**Per family**
Galagidae	*Galago senegalensis*	3	3	2.00 ± 0.00	6	0.00 ± 0.10	-
Lorisidae	*Nycticebus bengalensis*	1	6	-	25	0.39	0.58 ± 0.14
	Nycticebus coucang	1		-	5	0.39	
	Nycticebus pygmaeus	2		7.00 ± 1.41	14	0.60 ± 0.06	
	Perodicticus potto	2		2.00 ± 0.00	4	0.75 ± 0.38	
Lemuridae	*Eulemur fulvus*	2	7	2.50 ± 0.71	5	0.24 ± 0.11	0.28 ± 0.07
	Hapalemur griseus	1		-	1	0.67	
	Lemur catta	3		9.00 ± 1.73	27	0.17 ± 0.00	
	Varecia variegata	1		-	35	0.33	
Callitrichidae	*Callimico goeldii*	2	30	15.50 ± 13.44	31	0.00 ± 0.00	0.18 ± 0.06
	Callithrix geoffroyi	1		-	8	0.00	
	★*Callithrix jacchus*	12		20.15 ± 18.19	231	0.32 ± 0.11	
	Callithrix penicillata	1		-	16	1.00	
	Cebuella pygmaea	2		5.50 ± 3.54	11	0.00 ± 0.50	
	Leontopithecus chrysomelas	1		-	2	0.00	
	Leontopithecus rosalia	1		-	2	0.15	
	Saguinus labiatus	1		-	3	0.00	
	★*Saguinus oedipus*	9		4.75 ± 14.06	207	0.57 ± 0.11	
Cebidae	*Cebus capucinus*	2	25	10.00 ± 0.00	20	0.20 ± 0.13	0.30 ± 0.06
	Saimiri boliviensis	1		-	10	0.67	
	Saimiri sciureus	6		12.17 ± 10.09	73	0.11 ± 0.14	
	Sapajus apella	13		9.38 ± 2.18	122	0.33 ± 0.09	
	Sapajus nigritus	1		-	6	1.00	
	Sapajus xanthosternos	2		13.00 ± 12.73	26	0.21 ± 0.09	
Cercopithecidae	*Cercocebus galeritus*	4	107	3.50 ± 1.00	14	0.00 ± 0.24	0.23 ± 0.03
	Cercocebus torquatus	6		27.17 ± 32.46	163	0.11 ± 0.05	
	Cercopithecus mona	1		-	2	0.14	
	Cercopithecus wolfi	1		-	4	0.29	
	Chlorocebus aethiops	2		-	19	0.09	
	Colobus guereza	1		-	8	-0.22	
	Lophocebus albigena	2		7.50 ± 0.71	15	0.26 ± 0.02	
	Macaca arctoides	7		12.00 ± 9.13	84	0.12 ± 0.15	
	Macaca fascicularis	5		23.40 ± 12.20	117	-0.04 ± 0.11	
	Macaca fuscata	6		35.50 ± 46.28	213	0.28 ± 0.11	
	Macaca mulatta	45		42.27 ± 43.00	1902	0.26 ± 0.04	
	Macaca nemestrina	2		9.50 ± 6.36	19	0.15 ± 0.00	
	Macaca nigra	2		26.50 ± 20.51	53	0.60 ± 0.19	
	Macaca radiata	1		-	14	0.00	
	Macaca silenus	3		6.33 ± 0.58	19	0.42 ± 0.09	
	Macaca sylvanus	3		8.00 ± 0.00	24	-0.21 ± 0.31	
	Mandrillus leucophaeus	2		1.00 ± 0.00	2	-0.03 ± 0.00	
	Mandrillus sphinx	2		3.00 ± 1.41	6	0.33 ± 0.36	
	Papio anubis	1		-	7	0.00	
	Papio hamadryas	9		6.44 ± 2.46	58	0.52 ± 0.00	
	Papio papio	1		-	8	0.43	
	Semnopithecus priam	1		-	9	0.55	
Hylobatidae	★*Hylobates lar*	2	10	2.00 ± 0.00	2	0.07 ± 0.04	0.22 ± 0.18
	Hylobates moloch	4		8.50 ± 3.00	34	0.09 ± 0.13	
	Hylobates pileatus	1		-	36	0.33	
	Nomascus leucogenys	1		-	4	1.00	
	Symphalangus syndactylus	2		4.00 ± 2.83	8	0.00 ± 0.50	
Hominidae	*Gorilla gorilla*	18	60	6.61 ± 3.78	119	0.17 ± 0.07	0.22 ± 0.04
	Pan paniscus	2		11.00 ± 4.24	22	0.38 ± 0.25	
	Pan troglodytes	34		19.00 ± 27.54	646	0.24 ± 0.06	
	Pongo pygmaeus	6		16.17 ± 27.48	97	0.23 ± 0.12	
	All species pooled	248				0.23 ± 0.02	
