# Supplementary material for: A Metric-Based, Meta-Analytic Appraisal of Environmental Enrichment Efficacy in Captive Primates
Source: Animals (Basel). 2025 Mar 11;15(6):799. doi: 10.3390/ani15060799 (PMC11939658; doi:10.3390/ani15060799)
Supplement: Supplementary file 1 [file animals-15-00799-s001.zip › Duncan&Pillay_TableS4.pdf]

Supplementary: Table S4. *Summary of case reports in the literature relating to effects of environmental enrichment on captive primates and details of four protocols from environmental enrichment of animals in primate rehabilitation centre and sanctuary settings (termed 'Other reports' below). Enriched housing state was considered when environmental elements exceeded those needed for basic biological functioning and safety (i.e. food, water, enclosing barriers). Enrichment type was classified according to the specific aspects of biological functioning that the enrichment addressed. Enrichment access referred to how long the animals had access to or were administered the enrichment protocol and not the duration of the experimental treatment in which the enrichment was applied. Efficacy scores represent an index of efficacy ranging from -1 to 1, corresponding to an absolute deterioration and improvement in animal welfare respectively for all measures reported. An efficacy score of 0 implies no change in animal welfare status for all measures reported.*

	**Species**	**Institutional context**	**Number of subjects**	**Social conditions**	**Enclosure type**	**Housing state**	**Enrichment type**	**Enrichment access**	**Assessment method**	**Efficacy score**	**Citation**
Case reports	*Chlorocebus aethiops*	Laboratory	Undisclosed	Solitary	Standard housing	Impoverished	Social & spatial	1-3 days	Behavioural, Reproductive	0.25	[173]
	Papio hamadryas	Laboratory	1	Group housing	Indoor-outdoor	Enriched	Feeding	3-24 hours	Clinical	-1.00	[115]
	Gorilla gorilla	Zoo	7 - 12	Group housing	Outdoor	Undisclosed	Feeding (incidental)	>3 days	Behavioural	1.00	[118]
	Pan troglodytes	Laboratory	1	Solitary	Indoor	Enriched	Cognitive	>3 days	Behavioural, Clinical	0.67	[104]
	Pan troglodytes	Laboratory	1	Group housing	Undisclosed	Undisclosed	Training, social & spatial	Undisclosed	Behavioural, Clinical	1.00	[174]
	Macaca fascicularis	Laboratory	1	Solitary	Standard housing	Undisclosed	Object	Undisclosed	Clinical	-1.00	[114]
	Chlorocebus aethiops	Laboratory	1	Group housing	Indoor	Enriched	Feeding	Undisclosed	Clinical	-1.00	[116]
	Macaca mulatta	Laboratory	1	Solitary	Standard housing	Enriched	Social & object	>3 days	Behavioural, Clinical	1.00	[175]
Other reports	*Cercocebus torquatus*	Rehabilitation centre	69	Group housing	Outdoor	Enriched	Enclosure change	>3 days	Behavioural	0.00	[176]
	Cercocebus torquatus	Rehabilitation centre	69	Group housing	Outdoor	Enriched	Enclosure change	>3 days	Behavioural	0.25	[176]
	Nycticebus bengalensis + Nycticebus coucang	Rehabilitation centre	25 + 5	Mixed (some solitary, some social)	Mixed (some indoor, some outdoor)	Undisclosed	Feeding	>3 days	Behavioural	0.39	[77]
	Pan troglodytes	Sanctuary	28	Group housing	Indoor	Enriched	Enclosure change	>3 days	Behavioural	0.19	[177]
	Pan troglodytes	Sanctuary	7	Group housing	Indoor-outdoor	Enriched	Social	>3 days	Behavioural	0.50	[178]
