# Supplementary material for: A Metric-Based, Meta-Analytic Appraisal of Environmental Enrichment Efficacy in Captive Primates
Source: Animals (Basel). 2025 Mar 11;15(6):799. doi: 10.3390/ani15060799 (PMC11939658; doi:10.3390/ani15060799)
Supplement: Supplementary file 1 [file animals-15-00799-s001.zip › Duncan&Pillay_TableS5.pdf]

***Supplementary: Table S5*. Table of absolute-value beta-estimates and p-values from a generalised linear model (Captivity model) for contrasts between different types of environmental enrichment for captive primates. Values in bold indicate statistically significant differences.**

	Cognitive	*Combination*	*Enclosure change*	*Feeding*	*Interaction*	*Object*	*Olfactory*	*Other*	*Social*	*Training*	*Undisclosed*	*Visual*
Auditory	**β=0.47±0.18; *p*=0.012**	β=0.27±0.16; *p*=0.101	β=0.31±0.16; *p*=0.060	**β=0.34±0.16; *p*=0.032**	β=0.11±0.25; *p*=0.651	β=0.13±0.17; *p*=0.437	β=0.18±0.23; *p*=0.419	β=0.22±0.17; *p*=0.199	β=0.20±0.16; *p*=0.229	**β=0.44±0.17; *p*=0.011**	β=0.12±0.38; *p*=0.761	β=0.30±0.20; *p*=0.149
Cognitive		β=0.20±0.14; *p*=0.150	β=0.16±0.13; *p*=0.218	β=0.13±0.12; *p*=0.305	**β=0.58±0.23; *p*=0.012**	**β=0.33±0.15; *p*=0.024**	β=0.28±0.21; *p*=0.186	β=0.25±0.14; *p*=0.066	**β=0.27±0.13; *p*=0.044**	β=0.02±0.14; *p*=0.856	β=0.35±0.37; *p*=0.338	β=0.17±0.18; *p*=0.340
Combination			β=0.04±0.09; *p*=0.674	β=0.07±0.08; *p*=0.400	β=0.38±0.22; *p*=0.077	β=0.14±0.10; *p*=0.162	β=0.09±0.19; *p*=0.648	β=0.05±0.10; *p*=0.591	β=0.07±0.09; *p*=0.403	β=0.17±0.11; *p*=0.130	β=0.16±0.35; *p*=0.656	β=0.03±0.16; *p*=0.871
Enclosure change				β=0.03±0.07; *p*=0.671	**β=0.42±0.21; *p*=0.050**	β=0.17±0.09; *p*=0.069	β=0.12±0.19; *p*=0.509	β=0.09±0.10; *p*=0.340	β=0.11±0.08; *p*=0.192	β=0.13±0.11; *p*=0.215	β=0.19±0.35; *p*=0.584	β=0.01±0.16; *p*=0.947
Feeding					**β=0.45±0.21; *p*=0.030**	**β=0.20±0.09; *p*=0.029**	β=0.15±0.18; *p*=0.392	β=0.12±0.09; *p*=0.179	β=0.14±0.08; *p*=0.082	β=0.10±0.10; *p*=0.309	β=0.22±0.35; *p*=0.520	β=0.04±0.15; *p*=0.783
Interaction						β=0.25±0.22; *p*=0.266	β=0.30±0.27; *p*=0.265	β=0.33±0.22; *p*=0.134	β=0.31±0.21; *p*=0.140	**β=0.56±0.22; *p*=0.012**	β=0.23±0.40; *p*=0.564	β=0.41±0.25; *p*=0.099
Object							β=0.05±0.19; *p*=0.793	β=0.08±0.11; *p*=0.461	β=0.06±0.10; *p*=0.529	**β=0.31±0.12; *p*=0.013**	β=0.02±0.35; *p*=0.958	β=0.16±0.17; *p*=0.339
Olfactory								β=0.03±0.19; *p*=0.867	β=0.01±0.19; *p*=0.943	β=0.26±0.20; *p*=0.198	β=0.07±0.39; *p*=0.859	β=0.11±0.23; *p*=0.627
Other									β=0.02±0.10; *p*=0.860	β=0.23±0.12; *p*=0.058	β=0.10±0.36; *p*=0.777	β=0.08±0.16; *p*=0.626
Social										**β=0.24±0.11; *p*=0.029**	β=0.08±0.35; *p*=0.812	β=0.10±0.16; *p*=0.527
Training											β=0.33±0.35; *p*=0.358	β=0.15±0.17; *p*=0.383
Undisclosed												β=0.18±0.37; *p*=0.627
